# Prevalence and Correlates of Anemia among Adolescents Living in Hodeida, Yemen

**DOI:** 10.3390/children9070977

**Published:** 2022-06-29

**Authors:** Abdulghani Sulaiman Mohammed Al-Jermmy, Shadia Mohamed Idris, Ferima Coulibaly-Zerbo, Lara Nasreddine, Ayoub Al-Jawaldeh

**Affiliations:** 1 College of Medicine, University of Saba Region, Marib 14400, Yemen; abomarwan_1980@outlook.com; 2 College of Public and Environmental Health, University of Bahri, Khartoum 12217, Sudan; shadiaidris4@gmail.com; 3 World Health Organization (WHO), Sana’a 543, Yemen; zerbof@who.int; 4 Nutrition and Food Sciences Department, Faculty of Agriculture and Food Sciences, American University of Beirut, Beirut 11-0236, Lebanon; 5 Regional Office for the Eastern Mediterranean (EMRO), World Health Organization (WHO), Cairo 7608, Egypt; aljawaldeh@who.int

**Keywords:** anemia, prevalence, correlates, nutrition education, adolescents, Yemen

## Abstract

This study assesses the prevalence and correlates of anemia among adolescents living in the war-affected region of Hodeida in Yemen. A secondary objective was to examine the effect of a nutrition education intervention on hemoglobin levels among anemic adolescents. A cross-sectional study was conducted on a random sample of adolescents aged 15–19 years in Hodeida (*n* = 400). A questionnaire was administered to inquire about demographic, socioeconomic, lifestyle and clinical characteristics. Capillary blood was obtained, anthropometric characteristics were measured and stool samples were collected. As for the secondary objective, anemic adolescents were randomly assigned to an intervention group (nutrition education and iron supplementation) and a control group (iron supplements only). The prevalence of anemia was 37.8%. Female gender, khat chewing, excessive menstruation, and experiencing headaches, fatigue, or dizziness were independent predictors of anemia. In contrast, adolescents who attended private schools, and reported snack consumption or handwashing had a significantly lower risk of anemia. A sample of 116 adolescents participated in the intervention (3 months). Hemoglobin levels were significantly higher in the intervention group compared to the control. Our findings contribute to the identification of high-risk groups that should be targeted by context-specific interventions. The implemented multicomponent intervention may serve as a prototype for larger-scale preventive programs.

## 1. Introduction

Anemia is acknowledged as a global public health concern, especially in low- and middle-income countries [1,2]. Anemia is defined based on low concentrations of hemoglobin or a decrease in the number of red blood cells [2,3]. In clinical practice, it is categorized as mild, moderate or severe based on hemoglobin levels, with the cutoffs being based on age, sex and physiological state [4]. The decrease in hemoglobin concentrations that characterizes anemia would limit the transport of blood oxygen, rendering it insufficient to meet physiologic and metabolic needs [4]. This leads to diminished physical and mental capacity, while raising the risk of several other adverse health effects [2]. Anemia is recognized as a multifactorial condition, with a multifaceted etiology that intricately involves nutritional as well as non-nutritional factors and pathways [2]. Among the well-known causes of anemia, iron deficiency is unquestionably the most common, but other nutritional deficiencies can also contribute to anemia, including folate and vitamin B12 deficiencies [2,5,6]. In addition, there are numerous other non-nutritional factors that also cause anemia, such as inadequate access to water, sanitation, and hygiene; chronic exposure to infectious diseases; as well as inflammation and genetic hemoglobin disorders [2,5,6,7,8]. Non-health consequences of anemia comprise increased expenditure on health care and reduced propensity for income generation, thus carrying multiple impacts on the health and livelihoods of individuals, families and communities [2]. Acknowledging its deleterious impact at the individual and social levels, reduction in anemia has been identified as one of the World Health Assembly global nutrition targets for 2025 [9], and features in the Sustainable Development Goals list [10].

Adolescents are recognized as a vulnerable population regarding anemia [11]. This age group’s vulnerability is often attributed to the increased physiological demands for micronutrients (such as iron and folic acid) that accompany rapid physical growth, as well as to the micronutrient losses that may be caused by intestinal parasitic infestations, especially in developing countries [11]. Towards the end of adolescence, boys tend to replenish their nutrient stores, whereas girls continue to be vulnerable to anemia because of menstrual blood loss, and possibly gestation and lactation in the case of adolescent pregnancy, which is still common in several developing countries [12]. In adolescents, anemia may lead to impairments in growth, reduction in physical fitness and increased risk of infection [13,14,15]. In addition to its adverse impacts on physical growth and health status, anemia can also negatively affect cognitive development, school attendance and school performance [13,15], hindering their educational achievement as well as their future labor productivity [16]. In pregnant adolescents, anemia may raise the risk of maternal and neonatal mortality [17,18], delivery of low birth-weight infants, as well as birth complications [19]. Therefore, anemia does not only represent a concern for today’s adolescents, but also for the future development of communities and societies [20]. 

At the global level, the prevalence of adolescent anemia is estimated at 15%, but with large disparities between developed and developing countries (6% and 27%, respectively) [21]. In the Middle East region, the reported prevalence of anemia varies from 14% to 42% among children and adolescents [22]. The burden of anemia may be even worse in low-income countries and humanitarian settings, due to the severe disruptions in health and food systems [5]. In this context, the humanitarian crisis in Yemen continues to be among the worst in the world, perpetuated by conflict, economic collapse, diseases, and the breakdown of public services and institutions [23]. Shockingly, it is estimated that 80% of the population in Yemen requires humanitarian assistance [23]. Severe water shortages and associated poor hygiene, coupled with a collapse of sanitation systems, disruptions in the food system, and extensive population displacement have contributed to a significant rise in the spread of malnutrition and infectious diseases [24]. According to UN agencies, about half of Yemen’s population, estimated at about 26.8 million, live below the poverty line [25]. Since the 2015 intensification of the conflict in Yemen, the prevalence of malnutrition has increased at an alarming rate, with hunger and famine remaining a persistent threat in the country. The COVID-19 pandemic has also contributed to further aggravation and substantial increases in “the number of people living on the brink of starvation” [26]: two-thirds of the Yemeni population are currently reported to be food-deprived [27]. 

Previous studies have reported high prevalence rates of anemia in Yemen, reaching 40% among pregnant women and 49% among children [28,29]. However, to our knowledge, there are no studies that have examined the prevalence of anemia among adolescents in Yemen, or the factors that may be associated with anemia in this vulnerable population. It is in this context that this study was undertaken, with the aim of (1) assessing the prevalence of anemia among adolescents living in the war-affected region of Hodeida in Yemen; (2) investigating sociodemographic, clinical, and lifestyle-related factors that are associated with anemia in this population. A secondary objective of the study was to examine the effectiveness of a nutrition education intervention in improving hemoglobin levels and anemia-related nutrition knowledge among anemic adolescents in Hodeida. Data stemming from this study could help in the development of context-appropriate strategies and programs to address anemia, and its correlates, in Yemen.

## 2. Materials and Methods

### 2.1. Study Design and Sampling

A cross-sectional study was carried out in 2020 on a random sample of male and female adolescents aged 15–19 years attending secondary schools in the Hodeida Governorate of Yemen. Using a stratified multistage random sampling technique, 5 out of the 26 districts of Hodeida were randomly selected: Al-Hawak, Al-Hali, Al-Mina, Az-Zydiah and Az-Zuhrah. Based on the population size of 16,698 secondary school students enrolled in grades 10, 11, and 12 in these five districts (as per the Education Office of Hodeidah, Directorate of Statistics and Planning), the estimated sample size for the survey was estimated at 400, with 5% marginal error. The number of 15–19-year-old adolescent students to be selected from each district was determined using the proportional allocation method (based on the number of schools and students in each district). Within the identified districts and schools, subjects were selected randomly from student class lists, based on a systematic random sampling approach. Data collection was initially planned to be conducted in the school setting; however, due to the COVID-19 outbreak, schools were not operational in Yemen [30]. Therefore, the students who were randomly selected were invited to participate, and to meet with our researcher in the school facility, or any other location in the village, for data collection (based on the participants’ preferences). The response rate was 90%. 

Inclusion criteria were: participants must be aged between 15 and 19 years, and registered at a school in the Hodeida Governorate of Yemen. Adolescents were excluded from the study if they reported having a blood transfusion within 3 months prior to data collection; if they reported suffering from anemia caused by inherited or acquired diseases, or other medical conditions; if they reported to have taken treatments for anemia within 4 weeks prior to data collection; if they reported to be on medication that may interfere with appetite or nutrient absorption. Pregnant female adolescents were also excluded from the study. The study was approved by the Director of Education in Hodeida (25/1/2020). Further approval was obtained from the headmasters of the participating schools prior to data collection. The study was conducted in accordance with the Helsinki declaration, and informed consent was obtained from all research participants and their parents/guardians. 

### 2.2. Data Collection

A trained researcher conducted one-to-one interviews with the participating adolescents, using a pre-tested multicomponent questionnaire (Supplementary Material), in addition to measuring anthropometric characteristics, assessing blood hemoglobin concentrations, and obtaining stool samples. 

The multicomponent questionnaire inquired about the subject’s demographic and socioeconomic characteristics, such as age, gender, type of school (governmental vs. private), grade, father’s educational level and occupation, mother’s educational level and occupation, monthly family income, and crowding index (ratio of the number of people living in the household over the number of rooms in the household) [31]. The questionnaire also inquired about lifestyle characteristics among adolescents (smoking, physical activity, and khat chewing), hygiene-related habits (washing hands before eating, washing hands after eating, washing hands after using the toilet), clinical symptoms of anemia (headache, fatigue, dizziness, and shortness of breath, particularly with exertion), medical history (i.e., the presence of chronic diseases), and pubertal history (spermarche, Yes/No; menarche, Yes/No; number of pads per day). Information about number of meals per day, breakfast consumption (never, sometimes, always), consumption of snacks during the day (never, sometimes, always), intake of fresh vegetables (never, sometimes, always), and fast food (never, sometimes, always) was also obtained. In addition, the daily frequency of tea or coffee drinking (never vs. 1–3 times per day) and the timing of their consumption (immediately after or during a meal vs. after a period of time post meal) were evaluated. 

Anthropometric characteristics including weight (in kilograms) and height (in meters) were measured for all participating adolescents, using a stadiometer and a portable digital scale. Height measurements were obtained without shoes, and body weight was measured to the nearest 0.1 kg with the participant in light indoor clothing and with bare feet or stockings. Anthropometric measurements were interpreted using the World Health Organization (WHO) 2006 criteria [32] based on body mass index (BMI) Z-scores (BAZ) and height for age Z-scores (HAZ). BAZ was categorized into wasting (less than −2 standard deviation (SD)), normal (−2 to +1 SD), overweight or obese (greater than +1 SD). Adolescents were considered as stunted if their HAZ < −2. 

Point-of-care hemoglobin evaluation was conducted using the HemoCue 201 device. One drop of capillary blood was obtained from the participant’s fingertip using a sterile lancet, and placed into a microcuvette, which was later inserted into the electronic hemoglobin device after its calibration to zero. Previous studies have, in fact, validated HemoCue 201 against venous blood measures as the gold standard, and reported high predictability and sensitivity [33,34]. For instance, a 98.7% predictability for the HemoCue 201 device was reported by Levy et al. in a pediatric population, indicating a very high predictive power [33], while a sensitivity of 93% was reported by Yadav et al. [34] in a sample of pregnant women. In our study, anemia was defined based on hemoglobin sex-specific cutoff levels of 12g/dL for females and 13 g/dL for males, as defined by the WHO [4]. The severity of anemia was also identified based on the WHO categorization [4], as follows: for girls 15 years and older, mild anemia was defined as hemoglobin levels between 11 and 11.9 g/dL; moderate anemia as 8.0–10.9 g/dL and severe anemia as below 8.0 g/dL. For boys 15 years and older, mild anemia was defined as hemoglobin levels between 11 and 12.9 g/dL; moderate anemia as 8.0–10.9 g/dL and severe anemia as below 8.0 g/dL. 

The researcher also provided each participant with a plastic container for the collection of stools. Participants were asked to provide the stool sample after the interview, if possible, after which, the sample was taken immediately to the laboratory for analysis. Other participants provided the sample the second day after the interview. In total, 261 participants provided stool samples for the examination of parasites via the stool ova and parasites test. For this purpose, one drop of normal saline (0.85% NaCl) was placed on the glass slide and mixed with a thin smear of fresh stool. The smear was then examined using a calibrated microscope to detect ova and cysts, using the 20x and 40x objectives. The parasites, and their ova or cysts, were identified according to the shape, size, and internal structure characteristic of the species, which included *Entrobius vermicuaris, Entamoeba histolytica, Giardia lamblia, Ascaris lumbricoides, Schistosoma mansoni*, and others.

### 2.3. Intervention 

An intervention trial was conducted to test the impact of a nutrition education program among those who were anemic. Adolescents identified with anemia (*n* = 151) were asked to participate. Those who agreed to participate were randomly assigned into two groups: a control group that received iron supplements (Glovite capsule 200 mg) once daily for 3 months, and the intervention group, which received nutrition education and followed the same iron supplementation protocol as above. In addition, both the control and intervention groups were given two tablets of Mebendazole (deworming medication: Antiver 100 mg) per day for 3 days (six tablets in total), prior to the intervention. The educational sessions consisted of four sessions: (1) introducing anemia and the importance of iron, folate and vitamin B12 during adolescence; (2) explaining the risk factors, symptoms, signs, and, consequently, the complications of anemia; (3) providing information about iron-rich foods, enhancers and inhibitors of iron absorption, with a focus on khat chewing and its consequences on iron absorption; and (4) reviewing the previous sessions and providing the students with an educational pamphlet. 

A questionnaire exploring anemia-related nutrition knowledge was administered to the participants pre- and post-intervention. The questionnaire inquired about (1) the assessment of anemia; (2) the potential causes of anemia; (3) micronutrient deficiencies that may lead to anemia; (4) signs and symptoms of anemia; (5) foods that are rich in iron; (6) absorption of iron from plant-based vs. animal-based foods; (7) factors that may inhibit iron absorption and factors that may enhance iron absorption; and (8) whether anemia can be prevented and managed via the diet. For each participant, a knowledge score was computed based on the number of correct responses to the knowledge questions, with scores ranging from 0 to 12, the greater the score, the greater the knowledge. Hemoglobin levels were also measured before and after the intervention. During the 3-month period, thirteen anemic adolescents withdrew from this intervention (eight from the control group and five from intervention group), resulting in a study population of 103 adolescents. 

### 2.4. Statistical Analysis

Data were entered and analyzed using Stata (StataCorp. 2019. Stata: Release 16. Statistical Software. StataCorp LLC, College Station, TX, USA). A Shapiro–Wilk test was used to examine the normality of the data distribution of the continuous variables, with a *p*-value less than 0.05 indicating that the normality assumption of the data was violated. Anthropometrical Z-scores were calculated by Epi Info software. The sample was divided into anemic vs. non-anemic. Mann–Whitney U tests and Pearson’s chi-square tests were used for comparing differences in the distribution and proportions between anemic adolescents and those who were not anemic, respectively. Covariates were considered for the logistic regression if the *p*-value was less than 0.05 in the bivariate analysis. Multicollinearity of the covariates was assessed using variance inflation factors (VIFs); a VIF < 10 was considered to show no collinearity between the variables used in the model. Results from the logistic regression analyses are expressed as odds ratios (OR) or adjusted odds ratios (AOR) for anemia with 95% confidence interval (CI). 

For the intervention component, particularly for comparing the nutritional knowledge scores and hemoglobin levels at pre- and post-intervention, and between groups, paired *t*-tests and one-way ANOVA were used, respectively. A two-tailed *p*-value of less than 0.05 was considered statistically significant.

## 3. Results

### 3.1. Investigation of Anemia and Its Correlates 

**Figure 1:**
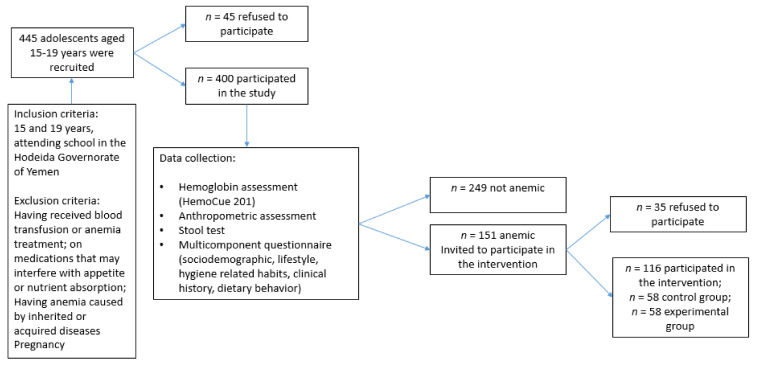
Recruitment and data collection from adolescent students aged 15–19 years attending secondary schools in the Hodeida Governorate of Yemen.

As shown in Figure 1, this study included 400 subjects aged between 15 and 19 years (mean age 16.9 years). Table 1 shows that more than half of the study population were females (56%) and living in urban areas (71%). Over two-thirds of the study population (68%) attended governmental schools. Only 33% and 12% of the adolescents’ fathers and mothers had completed secondary-level education or university, with almost 75% of the fathers being employed, compared to only 15% of the mothers. Approximately 40% of the participating adolescents belonged to families with incomes less than YER 30,000 (USD 50), with a comparable percentage (40%) reporting a crowding index of 4 and above (indicating a lower socioeconomic status). 

The mean hemoglobin level (±standard error (SE)) was 12.1 ± 0.1 g/dL, the median (interquartile range (IQR)) was 12.2 (2.1) g/dL, with a minimum of 7.1 g/dL and a maximum of 15.1 g/dL (data not shown). Based on hemoglobin levels, the prevalence of anemia was found to be 37.8% (*n* = 151). Among those with anemia, only one subject (female) had severe anemia (0.7%), 43% had moderate anemia (7 boys and 58 girls), and 56.3% had mild anemia (45 boys and 40 girls).

Table 1 shows that a higher percentage of females were anemic compared to males (44.4% vs. 29.4%) (*p* = 0.002). Moreover, anemia was more prevalent among those who lived in rural areas (*p* < 0.001), attended governmental schools (*p* < 0.001), and whose fathers and mothers completed primary or preparatory school (<0.001). Furthermore, significant differences were noted with regards to monthly family income, whereby 59% of those who belonged to families with low income (<YER 30,000) were anemic compared to 14.8% among those with an income > YER 70,000 (*p* < 0.001). 

Table 2 shows the lifestyle characteristics, dietary and hygiene-related habits of the study population by anemia status. The prevalence of anemia was significantly higher among those who reported khat chewing (46.6%) compared to those who did not (33.3%) (*p* = 0.010). Anemia prevalence was also significantly higher among adolescents who consumed only two meals (62.2%); those who never consumed breakfast (61.8%), snacks (47.1%), or vegetables (61.2%); and those who always consumed fast foods (52.2%) (*p* < 0.05). Adolescents who reported drinking tea after a period of time post meal were less likely to have anemia (26.5%) compared to those who reported to drink tea immediately after or during a meal (45.9%) (*p* < 0.001). One-third of adolescents who reported to wash their hands before eating were anemic, compared to 77.5% of adolescents who did not (*p* < 0.001).

Table 3 presents the participants’ medical and pubertal history, anthropometric characteristics, clinical symptoms, and stool test status (positive or negative for parasites) by anemia status. Anemia was significantly higher among those who experienced headaches, fatigue, dizziness, and shortness of breath, compared to those who did not report these symptoms (*p* < 0.001). Anemia was also significantly higher among those who had a positive stool test (61.7%) compared to those who had a negative test (38.3%) (*p* = 0.003). Moreover, anemia was more prevalent among those who experienced excessive menstruation (i.e., who reported using 3–5 pads per day) (*p* = 0.001) and among underweight (68.6%, as compared to 33.9% anemic adolescents with normal BMI status, *p* < 0.001) and stunted adolescents (57.8%, as compared to 35.2% anemic adolescents who were not stunted, *p* = 0.003).

### 3.2. Independent Predictors of Anemia among Adolescents

The results of the multivariable logistic regression analysis showed that being female (AOR: 17.0, 95% CI (2.6, 10.9)); khat chewing (AOR: 9.7, 95% CI (2.9, 32.8)); experiencing headaches (AOR: 8.4, 95% CI (3.7, 19.2)), fatigue (OR: 3.4, 95% CI (1.5, 7.4)), and/or dizziness (AOR: 5.1, 95% CI (2.1, 12.7)); and having excessive menstruation (OR: 8.1, 95% CI (1.6, 41.4)) were independent predictors of anemia among adolescents (Table 4). In contrast, the odds of having anemia were significantly lower among those who attended private schools (OR: 0.3, 95% CI (0.1, 0.7)) and those who reported the consumption of snacks (OR: 0.3, 95% CI (0.1, 0.7)), compared to those who attended governmental schools and never consumed snacks, respectively. Moreover, washing hands before eating was associated with lower risk of having anemia (OR: 0.2, 95% CI (0.1, 0.8)).

### 3.3. Intervention

For the intervention, the study population included 116 anemic adolescents, randomly sorted in the control (*n* = 58) or intervention group (*n* = 58). Mean scores of anemia-related knowledge and hemoglobin levels before and after the intervention are shown in Table 5 for both the control and intervention groups. Pre-intervention, there was no significant differences in knowledge, or in hemoglobin levels, between the two groups. 

Compared to the control group, those who received the nutrition education intervention had significantly higher knowledge scores (76.2 vs. 22.4 out of 100, *p* < 0.001) and hemoglobin levels (12.9 g/dL vs. 11.3 g/dL, *p* < 0.001) post-intervention. Moreover, among those who received the educational intervention, a significant difference was observed in their knowledge scores (76.2 vs. 21.7 over 100) and hemoglobin levels (12.9 g/dL vs. 10.5 g/dL) before and after the intervention.

## 4. Discussion

This study shows that 38% of adolescents in Hodeida, Yemen, were anemic in 2020, an estimate that highlights a public health concern of moderate significance as per the WHO criteria, but that is approaching the 40% threshold for classification as a severe public health problem [35]. The study also shows that female gender, heavy menstruation, and the practice of khat chewing were independent predictors of anemia, while snack consumption, washing hands before eating, as well as attending a private school were independently associated with lower risk of anemia. Clinical symptoms that were also found to predict anemia in the study population included headache, fatigue, and dizziness. A secondary objective of this study included the investigation of the effectiveness of a nutrition education intervention in improving hemoglobin levels and anemia-related knowledge among anemic adolescents. Our results show that the group of adolescents who had participated in the intervention had significantly higher hemoglobin levels and knowledge scores compared to the control, despite both groups receiving iron supplements for the trial period of 3 months. 

The prevalence of anemia in Yemeni adolescents (37.8%) is higher than that reported among 10–19-year-old adolescents in Nepal (31%) [36], and more than sevenfold higher than that reported among 12–16-year-old adolescents in Turkey (5.6%) [37]. When focusing on the female population, the prevalence of anemia among girls in Hodeida (44.4%) was higher than that observed among adolescent girls from Gaza (35.8%) [38], Dubai (18.1%) [39], and Turkey (8.3%) [37], while being lower than estimates reported from India (59.8%) and Oman (54%). It is important to note that the observed prevalence of anemia in Yemeni adolescents was higher than that previously reported by Al-Alimi et al. [40] among medical students in Hodeida University (30.4%). This is to be expected, since the study by Alimi-et al reported on iron deficiency anemia specifically, whereas in our study, the focus was on general anemia, which can be induced by several factors other than iron deficiency [2]. 

The fact that, in our study, girls were at a higher risk of anemia, is in line with results presented in the literature [36]. In general, adolescents are recognized as a population that is vulnerable to anemia [11], given the increased requirements of nutrition for growth, and, more specifically, the increased iron requirements for myoglobin in muscles and hemoglobin in the blood [41]. In girls, this is further exacerbated by the onset of menstruation [42]; in fact, our study shows that heavy menstruation is an independent predictor of anemia in adolescent girls. Other factors that may explain the observed gender disparities in the prevalence of anemia in Yemen include cultural and social factors that often disadvantage girls. For instance, gender inequality that may affect education, early marriage, and participation in household decision making may be important underlying determinants of malnutrition among adolescent girls, and potentially, their access to healthy diets and iron-rich foods [43]. Traditional cultural practices in some families in Yemen may also affect intra-household food distribution, prioritizing males at the expense of females [40]. In our study, girls had 17 times higher risk of having anemia compared to boys. This carries serious implications for adolescent girls’ health, whereby anemia can reduce their resistance to infection; impair physical growth and cognitive development; and decrease physical fitness, school performance, and work capacity [15,42,44,45]. In the case of adolescent pregnancy, which is common in Yemen [46], anemia may increase the risk of maternal and neonatal mortality, preterm labor, and low birth weight [47], thus perpetuating an intergenerational cycle of undernutrition. 

Khat chewing was also found to be an independent predictor of anemia in our study population. Because of its amphetamine-like stimulant effects [48], khat chewing has become an epidemic in Yemen, and a common practice among high school, college, and university students [40,49]. Previous studies have reported on the association between khat chewing and anemia, which could be explained by its potential impact on appetite [50]. In fact, some of the active substances in khat (such as cathinone) have appetite suppressant effects, acting on the central nervous system and causing delays in gastric emptying [51,52,53]. This anorexigenic effect may have repercussions on food intake, micronutrient adequacy, and hence, nutritional status [54]. In addition, khat contains a substantial amount of tannins, which can reduce the bioavailability of non-heme iron from plant-based foods, which represent the basis of the population’s diet in Yemen [55,56]. The WHO reported that khat chewing may cause dependency [57,58,59,60,61], while predisposing chewers to an array of serious medical conditions [48]. Raising awareness about the adverse health impacts of khat chewing, and the provision of medical and nutritional management that targets khat chewers, are important points for the prevention of undernutrition and anemia among adolescents in Yemen. 

This study also shows that some of the classic symptoms of anemia [62], including headaches, fatigue, and dizziness, were independent predictors adolescent anemia, being associated with an at least three times higher risk. These findings suggest that, in low-resource or humanitarian settings, where the regular assessment and detection of anemia may be challenging, a careful clinical evaluation of subjects may be adopted to identify anemia, or at least prioritize subjects for biochemical assessment [62]. It is important to acknowledge that this method would be more sensitive in detecting anemia of higher severity, rather than milder forms of anemia [62]. In addition, in agreement with findings from other studies [63,64], wasting and stunting, which are indicators of malnutrition, were associated with higher risk of anemia in the study population; however, these associations lost their significance after adjustment for other covariates. 

The current study shows that adolescents enrolled in private schools had a significantly lower risk of being anemic, and that the prevalence of adolescent anemia in governmental schools (48.2%) was three times higher than in private schools (15.6%). Students enrolled in governmental schools are usually from lower-income households and of lower socioeconomic status [65], the latter being recognized as an underlying determinant of anemia [40]. According to the WHO [2], it is crucial to consider socioeconomic factors within the determinants of anemia [2], and these include poverty, food insecurity, economic marginalization, access to social protection, and access to health care [2]. The pathways that may link these factors with anemia status are intricate, and often involve local sociopolitical processes and household-level environmental factors that can impact diet and micronutrient intake, as well as health and disease outcomes [66,67,68]. Recent estimates in Yemen suggest that more than 50% of the population lives below the poverty line, and suffer from a very low purchasing power, hence highlighting the crucial need for anemia reduction programs that tailor for the local socioeconomic context and challenges. 

In our study, the practice of washing hands was found to be independently associated with lower risk of anemia. Hygiene behaviors, such as handwashing, can in fact reduce the incidence of diarrheal diseases and respiratory infections, which could improve nutritional and micronutrient status [69]. It was also shown that handwashing decreases the risk of intestinal parasitic infections, which often lead to gastrointestinal inflammation, lower absorption of iron and other nutrients, and consequently undernutrition and anemia [70]. An intervention conducted among school-aged children in Ethiopia showed that the practice of handwashing decreased the risk of intestinal parasitic reinfection rates and significantly reduced anemia prevalence [71]. Interestingly, in our study, a positive stool test, indicating the presence of gastrointestinal parasites, was associated with higher risk of anemia, but this association lost its significance after adjusting for other covariates, which included handwashing. The multivariate regression model also included adjustments for socioeconomic status, a factor that was also previously shown to be associated with parasitic infections [72]. For instance, a study conducted in the city of Lubumbashi, Zaire, showed that the prevalence and intensity of *Ascaris lumbricoides* infection was lower in children of higher socioeconomic status [72], and a study conducted in the rural areas of Haryana showed an inverse relationship between helminthic infections and socioeconomic status among school-aged children [73]. This may explain the fact that the association between positive stool analysis and anemia lost its significance after adjustment for other covariates, including socioeconomic variables such as parental education, income, and type of school. There is a crucial need to promote and improve hygiene and other preventative programs in lower socioeconomic strata and low-resource settings in endemic countries such as Yemen, and align these programs with the 2030 WHO targets for parasite prevention and control, which include the elimination of parasite morbidity in school-aged children, and the establishment of efficient parasite control programs in adolescent girls [70]. 

Some dietary behaviors were found to be associated with the risk of anemia in our study. For instance, and in agreement with previous studies [74,75], regular fast food consumption was associated with higher risk of anemia, while regular vegetable consumption was associated with a lower risk. Similarly, meal patterns with regular breakfast consumption, three or more meals per day, and snack consumption were associated with lower risk of anemia in the study population. It is important to note that, in the multivariate model and after adjusting for the different covariates, snack consumption remained as the only significant dietary factor, being independently associated with a lower risk of anemia among adolescents. Snacking may, in fact, reflect a higher household food security status and greater food intake in general, which could be associated with higher micronutrient intake [76], and hence, a lower risk of anemia. Adolescents are, in fact, encouraged to consume up to three snacks per day in addition to the three main meals in order to meet their caloric and nutrient needs [77]. Another factor that was found to be associated with anemia was the timing of tea consumption, whereby drinking tea after a period of time post meal was associate with significantly lower risk of anemia among adolescents. This is in agreement with other studies, and is mostly explained by the fact that tea contains high amount of tannins, which can negatively influence iron absorption [78] and status. However, our study shows that the association between anemia and tea consumption loses its significance after adjustment for covariates, which included parental education levels, the latter being recognized as important modulators of dietary behavior and beverage intake among children [79]. 

A secondary objective of this study was to test the effectiveness of a nutrition education intervention on anemia-related knowledge and improving hemoglobin levels among anemic adolescents in Hodeida. Prior to the initiation of the intervention, both control and intervention groups were given a course of deworming medication to control intestinal parasitic infestations. In fact, in cases where intestinal parasitic infections are endemic, iron supplementation alone (or in combination with education) will not have the desired impact on anemia reduction [80,81] unless accompanied by proper deworming treatment. The trial consisted of a nutrition education intervention in addition to the provision of iron supplements. The literature suggests that educational interventions can achieve better rates of anemia reduction than the provision of supplements alone [2,82]. Our findings lend further support to this hypothesis, since the intervention group achieved significantly higher improvements in hemoglobin levels compared to the control group (12.9 g/dL vs. 11.3 g/dL). Nutrition education could improve an adolescent’s nutritional knowledge regarding iron-rich foods, which in turn leads to an improvement in hemoglobin levels [83]. The relative improvement in the intervention group was of 22.8% compared to only 5.6% in the control group, despite both groups receiving iron supplements. Nutrition education and improvement in dietary habits carry several advantages compared to other anemia prevention strategies, particularly in aspects related to sustainability and concomitant improvement in the intake of other nutrients [69]. The observed impact of the multicomponent intervention that was implemented in our study may serve as the basis for larger preventive and therapeutic programs targeting adolescents in Yemen.

This study has several strengths. It is, in fact, the first to report on the prevalence of anemia among adolescents in Yemen, a country that is shattered by war, poverty, and humanitarian emergencies. It is also the first to report on sociodemographic, lifestyle, and medical factors that are associated with anemia in this vulnerable population, which can help in the development of future preventive programs. However, the results of this study ought to be interpreted in light of the following limitations. First, the study was restricted to the region of Hodeida, which may limit the generalizability of the data to other areas in the country. Second, the biochemical assessment that was performed allowed for the detection of anemia, without assessing specific nutritional anemias, which limited our ability to report on the prevalence of iron deficiency anemia (or other micronutrient deficiencies that contribute to anemia, such as folate or vitamin B12). In addition, the method of anemia recognition was based on point-of-care hemoglobin estimation using the HemoCue 201 device, instead of complete blood counts. However, it is important to note that several studies have validated HemoCue 201 against gold standards in various populations [33,34,84] for the screening of anemia, and good correlation coefficients and high sensitivity and specificity were reported. The device has been widely used for community-based assessment of anemia among different age ranges [85], highlighting the practicality of the method and its high correlation with laboratory autoanalyzers. Furthermore, the dietary behavior questionnaire did not allow for detailed information on food consumption. For instance, although snack consumption was found to be associated with a lower risk of anemia in the study sample, we did not have information on the nature of these snacks. Additionally, like any interview-based study, the questionnaire collected data on self-reported behavior, which in some instances may differ from actual behavior [86]. However, and in order to minimize social desirability bias, the researcher was trained to decrease judgmental verbal and non-verbal communication with the study participants. Finally, it is important to acknowledge that the intervention arm that was conducted in our study was limited by several factors, including the fact that iron supplementation was used in anemic adolescents, although the exact nature or cause of anemia was not established. Despite its limitations, this study, may serve as a prototype for larger-scale preventive programs that include nutrition education, in addition to deworming and iron supplementation.

## 5. Conclusions

This study is the first to assess the prevalence of anemia and its correlates among adolescents in Yemen, an age group that is recognized as a nutritionally vulnerable population [11]. The study shows that approximately 38% of Yemeni adolescents were anemic in 2020, with girls having a 17-fold higher risk of being anemic compared to boys. The study also identified that higher socioeconomic status (i.e., attending a private school), better hygiene practices (i.e., washing hands before eating), and a greater food intake (i.e., snack consumption during the day) was associated with lower risk of anemia in the study population. In contrast, the practice of khat chewing, which is gaining an alarming popularity among young people, was associated with a 10-fold higher risk of anemia. Clinical symptoms that were also found to predict anemia in the study population included headache, fatigue, and dizziness, which suggests that in low-resource or humanitarian settings, such as in Yemen, a thorough clinical evaluation may be adopted to identify anemia, or at least prioritize subjects for biochemical assessment. Overall, the study findings contribute to the identification of high-risk groups that should be targeted by context-specific, integrated intervention programs to decrease the burden of anemia among the Yemeni adolescent population. The multicomponent intervention that was conducted in this study may serve as a prototype for larger-scale preventive programs that include nutrition education, in addition to deworming and iron supplementation. 

## Figures and Tables

**Table 1:** Sociodemographic characteristics of adolescents aged 15–19 years (*n* = 400) in Hodeida, Yemen, by anemia status.

	Total	Not Anemic	Anemic	*p*-Value ^1^
	*n* (%)			
	400 (100)	249 (62.3)	151 (37.7)	
Age (years) (mean ± SE)	16.9 ± 0.0	16.9 ± 0.1	16.9 ± 0.1	0.656
Age (years)				
15	22 (5.5)	15 (68.2)	7 (31.8)	0.794
16	130 (32.5)	84 (64.6)	46 (35.4)	
17	134 (33.5)	78 (85.2)	56 (71.8)	
18	102 (25.5)	64 (62.8)	38 (37.3)	
19	12 (3.0)	8 (66.7)	4 (33.3)	
Gender				
Male	177 (44.3)	125 (70.6)	52 (29.4)	**0.002**
Female	223 (55.7)	124 (55.6)	99 (44.4)	
Locality				
Urban	283 (70.8)	192 (67.8)	91 (32.2)	**<0.001**
Rural	117 (29.3)	57 (48.7)	60 (51.3)	
Type of school				
Governmental	272 (68.0)	141 (51.8)	131 (48.2)	**<0.001**
Private	128 (32.0)	108 (84.4)	20 (15.6)	
Grade				
10	152 (38.0)	101 (66.5)	51 (33.5)	0.082
11	132 (33.0)	72 (54.5)	60 (45.5)	
12	116 (29.0)	76 (65.5)	40 (34.5)	
Father’s education				
Primary, preparatory, or lower	267 (66.7)	147 (55.1)	120 (44.9)	**<0.001**
Secondary school or university	133 (33.3)	102 (76.7)	31 (23.3)	
Mother’s education				
Primary, preparatory, or lower	330 (82.5)	190 (57.6)	140 (42.2)	**<0.001**
Secondary school or university	49 (12.3)	59 (84.3)	11 (15.7)	
Father’s occupation				
Not employed	102 (25.5)	61 (59.8)	41 (40.2)	0.555
Employed	298 (74.5)	188 (63.1)	110 (36.9)	
Mother’s occupation				
Not employed	342 (85.5)	208 (60.8)	134 (39.2)	0.152
Employed	58 (14.5)	41 (70.7)	17 (29.3)	
Monthly Family Income (Yemeni Rial (YER)) ^2^				
<30,000	156 (39.0)	64 (41.0)	92 (59.0)	**<0.001**
30,000–70,000	129 (32.3)	87 (67.4)	42 (32.6)	
>70,000	115 (28.7)	98 (85.2)	17 (14.8)	
Crowding index				
≤2	84 (21.0)	52 (61.9)	32 (38.1)	0.198
2 to 4	156 (39.0)	105 (67.3)	51 (32.7)	
>4	160 (40.0)	92 (57.5)	68 (42.5)	

Abbreviations: SE: standard error. ^1^ *p*-values were calculated using chi-square tests and Mann–Whitney U tests, as applicable. ^2^ YER 1 = USD 0.00167. Numbers in bold are statistically significant (*p* < 0.05).

**Table 2:** Lifestyle characteristics, dietary habits, and hygiene-related habits among adolescents aged 15–19 years (*n* = 400) in Hodeida, Yemen, by anemia status.

	Total	Not Anemic	Anemic	*p*-Value
	*n* (%)			
	400 (100)	249 (62.3)	151 (37.7)	
**Lifestyle characteristics**				
Physical activity				
No	50 (12.5)	31 (62.0)	19 (38.0)	0.996
Sometimes	107 (26.8)	67 (62.6)	40 (37.4)	
Everyday	243 (60.8)	151 (62.1)	92 (37.9)	
Smoking				
No	343 (85.8)	211 (61.5)	132 (38.5)	0.458
Yes	57 (14.3)	38 (66.7)	19 (33.3)	
Khat chewing ^1^				
No	267 (66.7)	178 (66.7)	89 (33.3)	**0.010**
Yes	133 (33.3)	71 (53.4)	62 (46.6)	
**Dietary habits**				
Number of meals/day				
2	45 (11.3)	17 (37.8)	28 (62.2)	**<0.001**
3 or more	355 (88.7)	232 (65.4)	123 (34.6)	
Breakfast consumption				
Never	34 (8.5)	13 (38.2)	21 (61.8)	**0.001**
Sometimes	85 (21.2)	46 (54.1)	39 (45.9)	
Always	281 (70.3)	190 (67.6)	91 (32.4)	
Snack consumption				
Never	187 (46.7)	99 (52.9)	88 (47.1)	**0.001**
Sometimes	144 (36.0)	99 (68.8)	45 (31.2)	
Always	69 (17.3)	51 (7.9)	18 (26.1)	
Fast food consumption				
Never	80 (20.0)	59 (73.7)	21 (26.3)	**0.002**
Sometimes	228 (57.0)	146 (64.0)	82 (36.0)	
Always	92 (23.0)	44 (47.8)	48 (52.2)	
Fresh vegetable consumption				
Never	67 (16.8)	26 (38.8)	41 (61.2)	**<0.001**
Sometimes	263 (65.8)	166 (63.1)	97 (36.9)	
Always	70 (17.5)	57 (81.4)	13 (18.6)	
Tea drinker				
No	48 (12.0)	33 (68.8)	15 (31.3)	0.322
Yes	352 (88.0)	216 (61.4)	136 (38.6)	
Time of drinking tea ^2^				
Immediately after or during a meal	220 (62.5)	119 (54.1)	101 (45.9)	**<0.001**
After a period of time post meal	132 (37.5)	97 (73.5)	35 (26.5)	
Coffee drinker				
No	78 (19.5)	52 (66.7)	26 (33.3)	0.370
Yes	322 (80.5)	197 (61.2)	125 (38.8)	
Time of drinking coffee ^3^				
Immediately after or during a meal	203 (63.0)	119 (58.6)	84 (41.4)	0.218
After a period of time post meal	119 (37.0)	78 (65.5)	41 (34.4)	
**Hygiene-related habits**				
Washing hands before eating				
No	40 (10.0)	9 (22.5)	31 (77.5)	**<0.001**
Yes	360 (90.0)	240 (66.7)	120 (33.3)	
Washing hands after eating				
No	0 (0.0)	0 (0.0)	0 (0.0)	**
Yes	400 (100.0)	249 (62.3)	151 (37.7)	
Washing hands after using the toilet				
No	84 (21.0)	45 (53.6)	39 (46.4)	0.065
Yes	316 (79.0)	204 (64.6)	112 (35.40	

^1^ Fresh leaves of the khat tree (*Catha edulis* Forsk) chewed for their euphoric properties. ^2^ Assessed among tea drinkers, *n* = 352. ^3^ Assessed among coffee drinkers, *n* = 322. Numbers in bold are statistically significant (*p* < 0.05). ** Pearson’s chi-square test not applicable since 100% of the study population belong to one category; difference cannot be analyzed.

**Table 3:** Medical history, pubertal history, and anthropometric characteristics of adolescents aged 15–19 years (*n* = 400), in Hodeida, Yemen, by anemia status.

	Total	Not Anemic	Anemic	*p*-Value
	*n* (%)			
	400 (100)	249 (62.3)	151 (37.7)	
Medical History				
Presence of chronic diseases ^1^				
No	351 (87.7)	218 (62.1)	133 (37.9)	0.876
Yes	49 (12.3)	31 (63.3)	18 (36.7)	
Presence of Headache				
No	302 (75.5)	226 (74.8)	76 (25.2)	**<0.001**
Yes	98 (24.5)	23 (23.5)	75 (76.5)	
Presence of Fatigue				
No	316 (79.0)	221 (69.9)	95 (30.1)	**<0.001**
Yes	84 (21.0)	28 (33.3)	56 (66.7)	
Presence of Dizziness				
No	331 (82.7)	233 (70.4)	98 (29.6)	**<0.001**
Yes	69 (17.3)	16 (23.2)	53 (76.8)	
Presence of Shortness of Breath				
No	377 (94.3)	244 (64.7)	133 (35.3)	**<0.001**
Yes	23 (5.7)	5 (21.7)	18 (78.3)	
Stool test				
Negative	214 (82.0)	132 (61.7)	82 (38.3)	**0.003**
Positive	47 (18.0)	18 (38.3)	29 (61.7)	
Puberty History				
Menarche (among girls, *n* = 223)				
No	20 (9.0)	9 (45.0)	11 (55.0)	0.317
Yes	203 (91.0)	115 (56.6)	88 (42.4)	
Number of pads used/day (among girls that had experienced menarche)				
<3	187 (92.1)	112 (59.9)	75 (40.1)	**0.001**
3 to 5	16 (7.9)	3 (18.8)	13 (81.2)	
Spermarche				
No	11 (6.2)	5 (45.5)	6 (54.5)	0.055
Yes	168 (93.8)	122 (72.6)	46 (27.4)	
Anthropometric Characteristics				
BMI status ^2^				
Normal	342 (85.5)	226 (66.1)	116 (33.9)	**<0.001**
Wasting	35 (8.8)	11 (31.4)	24 (68.6)	
Overweight/Obese	23 (5.8)	12 (52.2)	11 (47.8)	
Stunted ^3^				
No	355 (88.8)	230 (64.8)	125 (35.2)	**0.003**
Yes	45 (11.2)	19 (42.2)	26 (57.8)	

Abbreviations: BMI, body mass index; HAZ, height for age Z-score; WHO, World Health Organization. ^1^ The presence of specific chronic diseases was also investigated, including the presence of rheumatic disease (1.2% of the total sample), peptic ulcers (2.3%), kidney disease (1%), bronchial disease (3%), diabetes (1.7%), and heart disease (0.5%). There were no significant differences between anemic and non-anemic individuals with respect to these diseases. ^2^ Anthropometry of adolescents was categorized based on WHO classification [32]. Within the overweight/obese category, there were 22 adolescents who were overweight and one who was obese. ^3^ Stunted if HAZ < −2, not stunted if HAZ ≥ −2. Numbers in bold are statistically significant (*p* < 0.05).

**Table 4:** Predictors of anemia among adolescents aged 15–19 years in Hodeida, Yemen (*n* = 400), based on logistic regression analysis.

Variables	Adjusted OR (95% CI)	*p*-Value
Age (years)	0.9 (0.7, 1.3)	0.785
Gender		
Male	1.0	
Female	**17.0 (2.6, 10.9)**	**0.003**
Locality		
Urban	1.0	
Rural	1.7 (0.7, 4.1)	0.222
Type of school		
Governmental		
Private	**0.3 (0.1, 0.7)**	**0.004**
Father’s education		
Primary or preparatory school, or lower	1.0	
Secondary school or university level	1.1 (0.5, 2.3)	0.905
Mother’s education		
Primary or preparatory school, or lower	1.0	
Secondary school or university level	0.4 (0.1, 1.3)	0.128
Monthly Family Income (Yemeni Rial (YER)) ^1^		
<30,000	1.0	
30,000–70,000	0.5 (0.2, 1.0)	0.052
>70,000	0.6 (0.2, 1.5)	0.281
Khat chewing ^2^		
No	1.0	
Yes	**9.7 (2.9, 32.8)**	**<0.001**
Number of meals/day		
2	1.0	
3 or more	0.4 (0.1, 1.1)	0.074
Breakfast consumption		
Never	1.0	
Sometimes	0.9 (0.2, 4.5)	0.908
Always	0.7 (0.2, 2.9)	0.595
Snack consumption		
Never	1.0	
Sometimes	**0.3 (0.1, 0.7)**	**0.006**
Always	0.4 (0.1, 1.1)	0.077
Fast food consumption		
Never	1.0	
Sometimes	1.3 (0.5, 3.5)	0.573
Always	2.2 (0.7, 6.5)	0.163
Fresh vegetable consumption		
Never	1.0	
Sometimes	0.6 (0.2, 1.6)	0.349
Always	0.5 (0.1, 1.7)	0.250
Tea drinking		
Immediately after or during a meal	1.0	
After a period of time post meal	0.7 (0.3, 1.4)	0.330
Washing hands before eating		
No	1.0	
Yes	**0.2 (0.1, 0.8)**	**0.016**
Presence of Headache		
No	1.0	
Yes	**8.4 (3.7, 19.2)**	**<0.001**
Presence of Fatigue		
No	1.0	
Yes	**3.4 (1.5, 7.4)**	**0.002**
Presence of Dizziness		
No	1.0	
Yes	**5.1 (2.1, 12.7)**	**<0.001**
Presence of Shortness of Breath		
No	1.0	
Yes	1.3 (0.3, 5.1)	0.739
Stool analysis for parasites		
Negative	1.0	
Positive	1.4 (0.5, 4.3)	0.526
Number of pads used/day (during menstruation)		
<3	1.0	
3 to 5	**8.1 (1.6, 41.4)**	**0.012**
BMI status ^3^		
Normal	1.0	
Wasting	2.1 (0.6, 7.9)	0.252
Overweight/Obese	2.3 (0.5, 10.1)	0.254
Stunted ^4^		
No	1.0	
Yes	1.5 (0.6, 4.0)	0.383

Abbreviations: BMI, body mass index; CI, confidence interval; HAZ, height for age Z-score; OR, odds ratio; WHO, World Health Organization. ^1^ YER 1 = USD 0.00167. ^2^ Fresh leaves of the khat tree (*Catha edulis* Forsk) chewed for their euphoric properties. ^3^ Anthropometry of adolescents was categorized based on WHO classification [32]. ^4^ Stunted if HAZ < −2, not stunted if HAZ ≥ −2. Numbers in bold are statistically significant (*p* < 0.05).

**Table 5:** Mean scores of anemia-related knowledge and hemoglobin levels before and after the intervention.

	Nutritional Knowledge Score			Hemoglobin Levels (g/dL)		
	Control Group (*n* = 50)	Intervention Group (*n* = 53)	*p*-Value ¹	Control Group (*n* = 50)	Intervention Group (*n* = 53)	*p*-Value ¹
	Mean ± SE			Mean ± SE		
Pre-intervention	21.9 ± 2.0	21.7 ± 1.9	0.945	10.7 ± 0.2	10.5 ± 0.2	0.379
Post-intervention	22.4 ± 1.6	76.2 ± 2.2	**<0.001**	11.3 ± 0.3	12.9 ± 0.1	**<0.001**
*p*-value ^2^	0.431	**<0.001**	--	**0.015**	**<0.001**	--

Abbreviations: SE, standard error. ¹ *p*-value comparing significant differences between control and intervention groups using an independent t-test. ^2^ *p*-value comparing significant differences within one group before and after intervention using a paired t-test. Numbers in bold are statistically significant (*p* < 0.05).

## Data Availability

Not applicable.

## Supplementary Materials

The following supporting information can be downloaded at: https://www.mdpi.com/article/10.3390/children9070977/s1, Questionnaire S1.
