# Supplementary material for: Prevalence and Correlates of Anemia among Adolescents Living in Hodeida, Yemen
Source: Children (Basel). 2022 Jun 29;9(7):977. doi: 10.3390/children9070977 (PMC9322810; doi:10.3390/children9070977)
Supplement: Supplementary file 1 [file children-09-00977-s001.zip › children-1705126-supplementary.pdf]

INDEX 1

Data collection sheet for cross-sectional survey

Serial Number: (   )
District: …………..   **Name of school:** ……………..
Type: **1. Public (   )**   **2. Private (   )**

I. Personal data:

1. **Age in years:**
2. **Gender**:   1. Male (   )   2. Female (   )
3. **Education year**: 1- 10 (   ) 2- 11 (   ) 3- 12 (   )
4. **Father's education**
   1. Illiterate (   )   2. Read and write (   )
   3. Primary or preparatory school (   )   4. Secondary school (   )
   5. University level (   )
5. **Father's Occupation:**
   1. Professional (   )   2. Non professional (   )
   3. Retired or without a job (   )   4. Farmer (   )
   5. Others (   )
6. **Mother's education**
   1. Illiterate (   )   2. Read and write (   )
   3. Primary or preparatory school (   )   4. Secondary school (   )
   5. University level (   )
7. **Mother's Occupation:**
   1. Professional (   )   2. Non professional (   )
   3. Retired or without a job (   )   4. Housewife (   )
   5. Others (   )
8. **Income:**
   1- Low (less than 30000)
   2- Medium (between 30000 – 70000)
   3- High (more than 70000)
9. **Crowding index** = No. of persons/ No. of room

II. General habit:

10. **Physical activity**: 1- No (   ) 2- Sometimes (   ) 3- Every day (   )
11. **Smoking:** 1- No (   ) 2- Sometimes (   ) 3- Every day (   )
12. **Khat chewing:** 1- No (   ) 2- Sometimes (   ) 3- Every day (   )

III. Daily foods practice:

13. **Number of meals/ day:** 1- One (   ) 2- Two (   ) 3- Three meals or more (   )
14. **Taking breakfast:** 1- No intake (   ) 2- always (   ) 3- Sometimes (   )
15. **Snacks intake:** 1- No intake (   ) 2- always (   ) 3- Sometimes (   )
16. **Fast foods intake:** 1- No intake (   ) 2- always (   ) 3- Sometimes (   )
17. **Fresh vegetables intake:** 1- No intake (   ) 2- always (   ) 3- Sometimes (   )
18. **Washing hands before food:** 1- Yes (   ) 2- No (   )
19. **Washing hands after food:** 1- Yes (   ) 2- No (   )
20. **Washing hands after toilet:** 1- Yes (   ) 2- No (   )
21. **Frequency of drinking tea / day:** 1- Do not drink (   ) 2- One to three times (   )
    3-Four times or more (   )
22. **Time of drinking tea:** 1- Immediately after or during intake meal (   )
    2- After a period of intake of meal (   )
23. **Frequency of drinking coffee / day:** 1- Do not drink (   ) 2- Once to three times (   )
    3- Four times or more (   )
24. **Time of drinking coffee:** 1- Immediately after or during intake meal (   )
    2- After a period of intake of meal (   )

IV. Medical History:

25. **History of chronic disease:** 1- Yes (   ) 2- No (   )
    If Yes.
26. **Type:** 1- Heart disease (   ) 2- Chest disease (   ) 3- Kidney disease (   )
    4- D.M (   ) 5- Others (   )

V. Puberty History:

For Females:

27. **Do you experience menarche?** 1- Yes (   ) 2- No (   )
    If yes:
28. **Duration of menstruation:** 1- Less than four days (   ) 2- four to seven days (   )
    3- More than seven days (   )
29. **Amount (number of towel/day):** 1- Less than three pads (   )
    2- From 3-5 pads (   )
    3- More than five pads (   )

For Males:

30. **Do you experience speromarche?**
    1- Yes (   ) 2- No (   )

VI. Symptoms related to anemia:

31. **Symptoms of anemia:**
    1- Headache (   ) 2- Fatigue (   ) 3- dizziness (   )
    4- Shortness in breathing particularly with exertion (   )
    5- Loss concentration (   )

VII. Anthropometric measurements:

32. Weight: …………kg
33. Height: …………..meters

VIII. Laboratory Examination:

Stool examination

34. Parasite 1- positive ( ) 2- negative ( )
If positive
35. Type:

1. Ascaris Lumbricid ( ) 2. Trichuris trichura ( ) 3. Hymenopepis nana ( )
4. Ancylostoma ( ) 5. Schistoma mansoni ( ) 6. Enterbius ermicularis ( )
7. Entomoeba histolytica ( ) 8. Ciardia lamblia ( ) 9. Other ( )

IX. Blood Examination:

36. Hb= gm/dl

INDEXIII

Data collection sheet for the intervention study

Name: **Age:**

Sex: 1. Male ( ) 2. Female ( )

Adolescent's condition pre treatment:

- Hb: gm/dl
- Symptoms:
  1- Breathing difficulty ( ) 2- Tired/weakness ( )
  3-. Dizziness ( ) 4- Headache ( )
- Palmer pallor: 1. Yes ( ) 2. No ( )
- Palpitation 1. Yes ( ) 2. No ( )
- Conjunctiva pallor 1. Yes ( ) 2. No ( )
- Scholastic achievement (grades of mid years examination)
  1. Poor ( ) 2- Good ( )
  3. Very good ( ) 4- Excellent ( )

Adolescent's condition post treatment:-

- Hb: gm/dl
- Symptoms:
  1- Breathing difficulty ( ) 2- Tired/weakness ( )
  3- Dizziness ( ) 4- Headache ( )
- Palmer pallor: 1. Yes ( ) 2. No ( )

- Palpitation 1. Yes ( ) 2. No ( )
- Conjunctiva pallor 1. Yes ( ) 2. No ( )
- Scholastic achievement (grades of mid years examination)

  1. Poor ( ) 2- Good ( )

  3. Very good ( ) 4- Excellent ( )

Compliance with treatment:

1. Complete ( ) 2- incomplete ( ) 3- non compliance ( )

Adolescent's Knowledge toward anemia

1- Heard about anemia: 1- Yes ( ) 2- No ( )

2- Anemia is a serious condition: 1- Yes ( ) 2- No ( ) 3-unsure ( )

3- Anemia effects on growth and scholastic achievement 1- Yes ( ) 2- No ( ) 3-unsure ( )

4- In anemia there is? 1- Increased red blood cells ( ) 2- Decreased hemoglobin ( ) 3- Increased hemoglobin ( ) 4- Don't know ( )

5- Nutrient deficient in anemia: 1- Iron ( ) 2- Calcium ( ) 3. Iodine ( ) 4- Don't know ( )

6- Causes of anemia: 1- Worm infestation ( ) 2- Poor diet ( ) 3- Excessive bleeding ( ) 4- All three are correct ( ) 5- Don't know ( )

7- Signs and symptoms of anemia: 1- Tiredness/body weakness ( ) 2- Conjunctiva pallor ( ) - 3- Palmer pallor ( ) 4- breathing difficulty ( ) 5- Reduced physical activity ( ) 6- dizziness ( ) 7- All are correct ( ) 8- Don't know ( )

8- Iron-rich food: 1- Green leafy vegetables ( ) 2- Meat, poultry 3- All are correct ( ) 4- Don't know ( )

9- Iron in meat is absorbed at the same rate as iron in a plant food: 1- Yes ( ) 2- No ( ) 3- Don't know ( )

10- Do we include iron-rich food in daily diet: 1- Yes ( ) 2- No ( )

11- Factors such as coffee and tea inhibit iron absorption: 1- Yes ( ) 2- No ( ) 3- Don't know ( )

12- Factors such as vitamin C enhances iron absorption: 1- Yes ( ) 2- No ( ) 3- Don't know ( )

13- It's possible to prevent and treat anemia by foods: 1- Yes ( ) 2- No ( ) 3- unsure ( )
